# Remotely Sensed Imagery for Early Detection of Respiratory Disease in Pigs: A Pilot Study

**DOI:** 10.3390/ani10030451

**Published:** 2020-03-09

**Authors:** Maria Jorquera-Chavez, Sigfredo Fuentes, Frank R. Dunshea, Robyn D. Warner, Tomas Poblete, Rebecca S. Morrison, Ellen C. Jongman

**Affiliations:** 1 Faculty of Veterinary and Agricultural Sciences, University of Melbourne, VIC 3010, Australia; sigfredo.fuentes@unimelb.edu.au (S.F.); fdunshea@unimelb.edu.au (F.R.D.); robyn.warner@unimelb.edu.au (R.D.W.); tomas.poblete@unimelb.edu.au (T.P.); 2 Research and Innovation, Rivalea (Australia) Pty. Ltd., Corowa, NSW 2646, Australia; RMorrison@rivalea.com.au; 3 Animal Welfare Science Centre, Faculty of Veterinary and Agricultural Sciences, University of Melbourne, Parkville, VIC 3010, Australia; ejongman@unimelb.edu.au

**Keywords:** animal monitoring, imagery, computer vision, animal health, symptoms, physiological changes

## Abstract

**Simple Summary:**

Respiratory disease in pigs causes suffering in infected animals and economic losses to producers. One of the most appropriate approaches to minimising these negative effects is by the early detection of infected animals. This pilot study aimed to use computer-based techniques to measure changes in temperature (eye and ear-base temperature), heart rate and respiration rate of pigs from thermal-infrared and conventional images. These measures, together with clinical observations, were obtained from pigs that were infected with *Actinobacillus pleuropneumoniae* (APP) and from pigs that were healthy. Infected pigs showed higher temperature and heart rate than healthy pigs across the period analysed. Respiration rate showed less difference between infected and healthy pigs. In addition, the biggest changes in these measures were recorded from six hours before the clinical observations identified sick animals. Results have highlighted that computer vision techniques can provide important and useable data regarding physiological changes that can indicate early signs of respiratory infection in pigs. This could aid the management of the disease, increasing the success of the treatment and decreasing the rate of severe cases and death.

**Abstract:**

Respiratory diseases are a major problem in the pig industry worldwide. Due to the impact of these diseases, the early identification of infected herds is essential. Computer vision technology, using RGB (red, green and blue) and thermal infrared imagery, can assist the early detection of changes in animal physiology related to these and other diseases. This pilot study aimed to identify whether these techniques are a useful tool to detect early changes of eye and ear-base temperature, heart rate and respiration rate in pigs that were challenged with *Actinobacillus pleuropneumoniae.* Clinical observations and imagery were analysed, comparing data obtained from animals that showed some signs of illness with data from animals that showed no signs of ill health. Highly significant differences (*p* < 0.05) were observed between sick and healthy pigs in heart rate, eye and ear temperature, with higher heart rate and higher temperatures in sick pigs. The largest change in temperature and heart rate remotely measured was observed around 4–6 h before signs of clinical illness were observed by the skilled technicians. These data suggest that computer vision techniques could be a useful tool to detect indicators of disease before the symptoms can be observed by stock people, assisting the early detection and control of respiratory diseases in pigs, promoting further research to study the capability and possible uses of this technology for on farm monitoring and management.

## 1. Introduction

The pig industry continues to face the presence of respiratory diseases, such as pleuropneumonia [1,2]. The pathogens that cause these diseases are often found in commercial piggeries where there are large number of animals housed together [1]. In the case of pleuropneumonia infections, these infections are known to easily propagate across young pigs (8–16 weeks of age) and generate clear symptoms from 11 to 23 h after the animal is artificially infected [3]. Some of the symptoms that can be observed are lethargy, high body temperature, abdominal breathing and vomiting. Respiratory infections lead to various issues, affecting not only the wellbeing of animals, but also the pig industry by increasing the cost of production and the use of medication programs to treat the disease [1,4,5,6,7]. A range of antimicrobials are used in the treatment of sick animals, administered by food and/or water, or individually dosed by injection and orally [6,7]. Although the use of antimicrobials has benefits and can significantly improve welfare and decrease the mortality rates in pigs, their excessive and inappropriate use has become a concern because of the emergence and rise of antimicrobial resistance (AMR) in animals and humans, promoting measures to control and decrease antimicrobial use in livestock [8,9].

Due to the economic impact on the swine industry, the impact on the wellbeing of pigs, and the global call for the prudent and targeted use of antibiotics, the early identification of respiratory diseases is essential. Non-invasive methods have been investigated to detect illness in several animals. For example, microphones are being used to assess coughing sounds as a warning of developing outbreak of respiratory infections [10,11,12]. Moreover, computer vision techniques have been increasingly studied to evaluate their potential to detect several illnesses in animals. For instance, Schaefer et al. [13] reported that skin temperatures of calves obtained from thermal infrared (TIR) cameras were useful to detect viral infections even some days before the clinical scores indicated signs of illness. In addition, this technology has also been used to detect fever in pigs [14]. TIR imagery has also been implemented to assess respiration rate (RR) by manually counting the changes of temperature and colour observed around the affected cows’ noses [15].

Moreover, computer algorithms able to analyse RGB (red, green and blue) imagery to assess animal behaviour and liveweight among others [16,17] are being investigated as tools to assess health and welfare in pigs. Processing of RGB imagery has also been used for human monitoring, which has shown promising results when evaluating physiological parameters in people [18,19]. For example, Poh et al. [20] assessed heart rate (HR) of people using RGB videos and computer algorithms, which tracked human faces and detected changes on luminosity related to cardiac pulse. In addition, Zhao et al. [19] reported positive results when analysing HR and RR of people from RGB videos. Their study showed correlation coefficients (r) between 0.95 and 0.99 (*p* < 0.001) when analysing the relation between HR and RR measurements obtained from videos and an OmniPlex^®^ data acquisition system (Plexon, Inc., Dallas, TX, USA). Although the implementation of these techniques is less advanced in animals than in humans, some researchers are attempting to develop techniques able to remotely assess physiological parameters in animals. For instance, Weixing and Zhilei [21] studied the possible implementation of computer algorithms to measure RR of pigs from RGB videos.

The present study showed that changes in eye temperature, ear-base temperature, HR and RR, remotely measured could indicate a pig’s illness before clinical signs can be detected by technicians. Therefore, this pilot study evaluated the possible implementation of imagery and developed computer algorithms to remotely identify early signs of *Actinobacillus pleuropneumoniae* (APP) infection in pigs.

## 2. Materials and Methods

### 2.1. Animals and Sample Collection

This pilot study was part of a larger project that involved a disease challenge and investigation of new vaccine for APP. All animal procedures had prior institutional ethical approval (Protocol ID: 17V060C) under the requirement of the New South Wales Prevention of Cruelty to Animals Act (1979) [22] in accordance with the National Health and Medical Research Council/Commonwealth Scientific and Industrial Research Organisation/Australian Animal Commission Australian Code of Practice for the Care and Use of Animals for Scientific Purposes [23].

As part of the larger study, a total of 46 weaned pigs, weaned at 24 days of age, were moved to the shed and divided into four pens (5 × 3.3 m) at 9 weeks of age. All commercial weaner, grower/finisher diets were fed ad libitum during the experiment. Three of these groups (14, 14 and 13 pigs respectively—floor space 1.1 m^2^/pig) were vaccinated against *Actinobacillus pleuropneumoniae* (APP), and one group (5 pigs—floor space 3.3 m^2^/pig) did not receive any vaccine. Pigs were challenged at 14 weeks of age with APP infection via intranasal atomiser at 2 mL 1 × 10^10^. After this challenge, they were regularly monitored over the next ten days (initially monitored every 2–3 h, and then the frequency was modified according to the condition of pigs) and remained in the shed until 24 weeks of age.

Clinical observations, TIR images and RGB videos were recorded at regular intervals before pigs were challenged with APP and the day after the challenge. On the day of the challenge, the footage was recorded approximately two hours before pigs commenced being inoculated intranasally with APP. The rest of the footage was obtained the next day, during the same periods when clinical observations were collected (7:30; 10:30; 12:30; 14:30). During this period, the mean temperature was 12 °C, the mean relative humidity was 71% and the mean wind speed reached 13 km/h.

In the present study, clinical observations, which have been linked to APP infections in previous studies [3,24], were included in a scoring system to identify animals with signs of this disease (Table 1). This scoring system was used by skilled stock people, who assigned scores from 0 to 3 when evaluating nasal discharge, coughing, laboured breathing, reddened conjunctiva, lethargy, anorexia and fever (rectal temperature).

When an animal obtained a total score of 3 or more or exhibited a rectal temperature above 40 °C, it was considered to be showing clinical symptoms of APP, labelled “sick” and treated. The animals that did not show any symptoms of APP (score 0) were labelled “healthy”.

TIR and RGB images of animals were simultaneously recorded for 1 min while lying or standing in the home pen by holding the device about 1 m away. These images were collected just before the challenge and during 4 periods the next day (7:30; 10:30; 12:30; 14:30). TIR images were used to assess eye temperature and ear-base temperature, non-radiometric infrared videos were used to assess RR, and RGB videos were used to assess HR.

### 2.2. Cameras Used and Image Processing

An integrated camera system was used. This consisted of a visible RGB video camera and a TIR camera connected to a web-based system which can simultaneously capture and store the videos and TIR images to be downloaded for analysis. The TIR camera (FLIR AX8; FLIR Systems, Wilsonville, OR, USA) had a spectral range of 7.5–13 μm, accuracy of ±2 °C, and emissivity of 0.985, and a frame rate of 1 image per second. The RGB video camera was a Raspberry Pi Camera Module V2.1 (Raspberry Pi Foundation, Cambridge, UK), which has an 8-megapixel sensor with a resolution of 640 × 360 pixels, and a frame rate of 30 frames per second. In addition, a FLIR^®^ ONE Thermal Imaging Case (FLIR Systems, Wilsonville, OR, USA) attached to an iPhone^®^ 5 (Apple Inc., Cupertino, CA, USA) was used to record non-radiometric IR videos.

**Figure 1:**
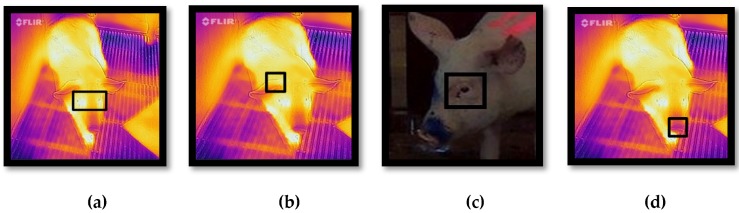
Regions of interest (ROIs) for image processing; (**a**) eye area (for eye-temperature), (**b**) base of ear (for ear-base temperature), (**c**) eye area (for heart rate) and (**d**) nose area (for respiration rate).

Collected images were processed using customised algorithms developed in Matlab^®^ R2018b (Mathworks Inc., Natick, MA, USA), which are described by Jorquera-Chavez et al. [25]. In the case of TIR images, this algorithm firstly extracted the radiometric information of each image, by using FLIR^®^ Atlas SDK [25,26]. Secondly, it allowed the selection of the eye and ear-base as region(s) of interest (ROI(s)), from where the temperature were extracted (Figure 1a,b). The selection of eye and ear-base areas as ROIs in this study was based on studies that have shown these areas to be more practical and accurate when using TIR images to measure body temperature [14,27].

RGB videos were processes by the respective algorithm, which identifies changes in luminosity in the green colour channel within the ROI (eye area; Figure 1c) and applies a second-order Butterworth filter and a fast Fourier transformation (FFT) after the signal has been obtained. To improve the accuracy of this analysis, the ROI was tracked through a computer vision algorithm following the methodology suggested by Jorquera-Chavez et al. [25]. This methodology first selects the most representative features within the ROI by using different pattern recognition techniques which then are tracked along with the sequential frames of the video. The eye area was used as ROI because it presents low density hair, and because this area has been shown to be useful when using imagery in humans and animals [14,28].

Furthermore, for the analysis of respiration rate, non-radiometric infrared videos were processed using an algorithm that identifies the changes in pixel intensity values within the ROI (nose area; Figure 1d), which are related to the air exchange during exhalation and inhalation [25].

### 2.3. Data Analysis

From the data set collected, the pigs that had measurements at all 4 time periods (7:30; 10:30; 12:30; 14:30) were selected to be included in the analysis. Considering only these animals, a comparison was made between the data from “sick” animals (score ≥ 3; *n* = 5) and the data from “healthy” animals (score = 0; *n* = 6). The data management and analysis were conducted in Microsoft Excel and Genstat^®^ for Windows 18th Edition (VSN International, Hemel Hempstead, UK).

Means of the physiological parameters remotely obtained (eye temperature, ear-base temperature, heart rate and respiration rate) were compared between groups at each of the 4 time points. Repeated-measures ANOVA was performed in order to test the group by time interaction in addition to the main effects. Plots of residuals vs fitted values were evaluated to assess the assumption of constant variance. The least significant difference (LSD) was used to test whether these physiological parameters were significantly different between “sick” and “healthy” pigs. Furthermore, the data obtained from clinical observations and computer vision techniques were also compared in order to identify whether remotely obtained parameters could detect physiological changes in pigs affected by respiratory disease before clinical signs were evident. To do this, the time at which the physiological parameters became significantly different between “sick” and “healthy” animals was compared to the time point when pigs where labelled “sick” according to clinical observations (score ≥ 3).

## 3. Results

The differences in temperature, heart rate and respiration rate shown by “sick” and “healthy” pigs across all 4 time points on the day after the challenge are presented and compared below. The repeated-measures ANOVA indicated significant effects of time × treatment interaction in all four remotely measured physiological parameters.

**Figure 2:**
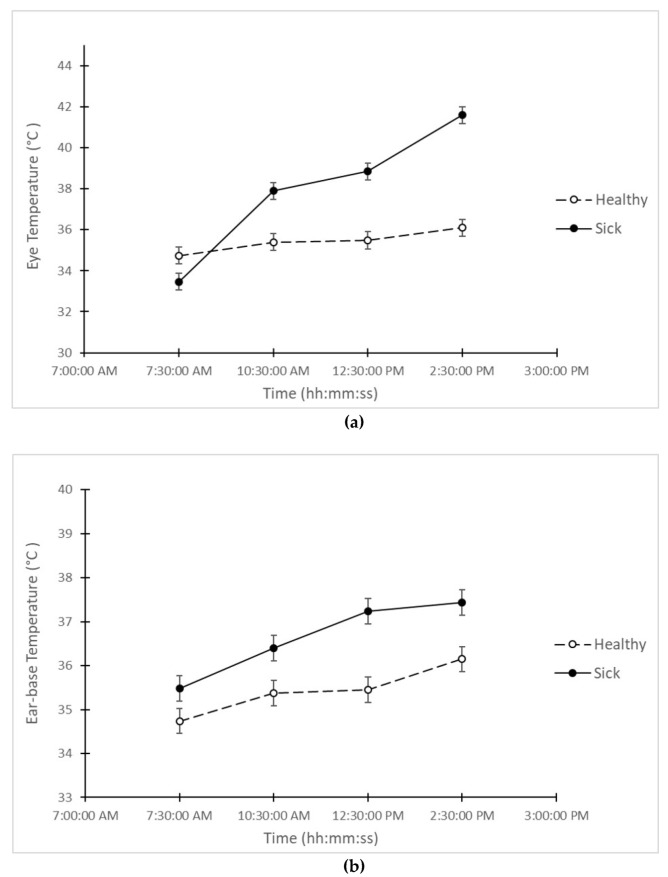
Average measurements of temperature (degrees Celsius) in “sick” and “healthy” animals at all 4 time points on the day after the challenge. On (**a**) eye temperature. On (**b**) ear-base temperature.

Both eye and ear-base temperatures had a greater increase in “sick” pigs than in “healthy” pigs (see Figure 2). In the case of eye temperature, the average temperature in the “sick” group increased 8.1 °C from 7:30 to 14:30 the day after they were challenged, while the average of eye temperature of the “healthy” group increased 1.4 °C during the same period. In addition, ear-base temperature appeared to be on average 0.8–1.8 °C higher in “sick” than in “healthy” pigs across the 4 time points measured on the day after the challenge. Furthermore, the differences in eye and ear-base temperature between “sick” and “healthy” animals were significantly higher (*p* < 0.05) than the LSD (0.84 and 0.6 respectively) across the four periods (7:30; 10:30; 12:30; 14:30).

**Figure 3:**
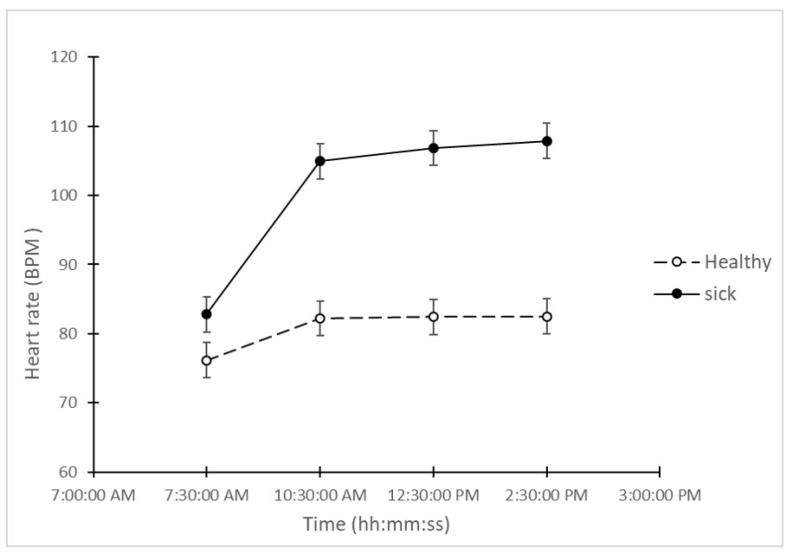
Average measurements of heart rate (beats per minute) in “sick” and “healthy” animals at all 4 time points on the day after the challenge.

Heart rate also showed larger changes in the “sick” pigs than in the “healthy” pigs, when comparing the measurements at all 4 time periods (Figure 3). The average HR of the “sick” group increased from 82.8 BPM at 7:30 to 107.9 BPM at 14:30. On the other hand, the average HR of the “healthy” group only increased from 76.2 to 82.5 during the same period. In addition, the difference of HR between “sick” and “healthy” animals increased from 6.62 to 25.37 (BPM) between 7:30 and 14:30, showing significant differences across all time points (LSD = 5.46; *p* < 0.05).

**Figure 4:**
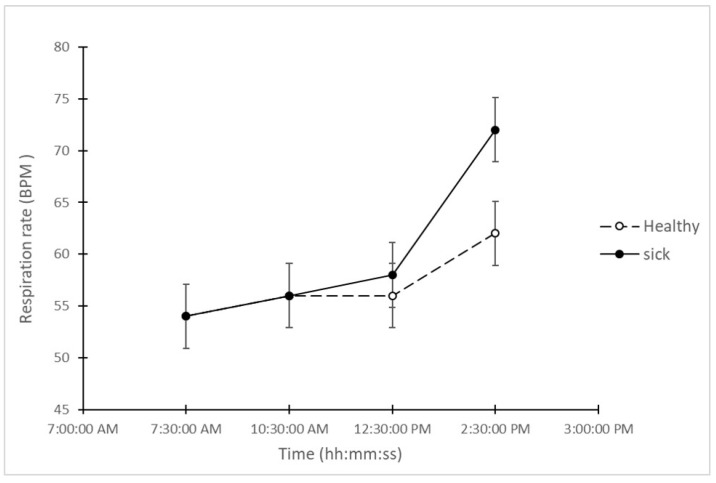
Average measurements of respiration rate (breath per minute) in “sick” and “healthy” animals at all 4 time points during the day after the challenge.

As shown in Figure 4, the respiration rate remotely obtained showed a different pattern to temperature and heart rate. Respiration rate measures of both groups (“sick” and “healthy”) were similar at the two first time points (54–56 breath per minute). At the third time point (12:30 p.m.), the average respiration rate of “sick” animals slightly differed to the average respiration rate of “healthy” animals (2 breath per minute higher in the “sick” group). The difference between groups increased in the last period recorded, where the average of respiration rate in the “healthy” group was 62 breath per minute, while the “sick” group reached in average 72 breath per minute. The comparison between the respiration rate of these two groups showed a significant difference only at the last time point (LSD = 6.22; *p* < 0.05).

## 4. Discussion

Respiratory disease in pigs is linked to production losses and a decrease in animal wellbeing and is considered as one of the most significant challenges for the pig industry [1]. The morbidity rate of respiratory disease in pigs has been described to be between 30% and 70% [1]. Commercial pigs normally encounter stress factors that increase their susceptibility to infectious pathogens such as APP, which can also decrease growth and feed intake [3]. In addition to the negative effects that these infections have on the animal and the pig industry, the concern about the amount of antibiotics that are used to treat ill pigs has promoted the search of technology to allow producers to identify disease early so targeted management and health programs can be implemented [29,30].

Monitoring tools for early detection of respiratory infection are a key factor to control these diseases and decrease their impact on animal production and welfare. Moreover, real time and individual monitoring could assist the early and individual identification of sick animals, allowing the administration of antimicrobials in the early stages of onset of disease and only when the animal is ill, and with the appropriate doses and period of time. The findings from this study suggest the possible implementation of computer vision techniques to detect physiological changes in sick animals at an early stage.

When analysing TIR images for remote temperature, eye area and ear base were used as ROIs due to the positive results these areas have shown when comparing temperatures from TIR images and rectal or vaginal temperature [14,31]. Eye temperature has been reported as a good indicator of core body temperature, which appears not to be affected by ambient temperature [14,32]. Ear-base temperature has also been identified as a good indicator of core body temperature and has been suggested to be easier to record from different positions [14,31]. Similar to the current study, the study by Schaefer et al. [13] compared clinical scores and temperatures obtained from TIR images for detecting early signs of bovine viral diarrhoea virus (BVDV) in calves. They observed that relevant changes in eye temperature started from day one after calves were inoculated with the infective agent. Moreover, they reported that clear changes in temperatures remotely obtained occurred several days before clinical observations were identified in sick animals. Those results are consistent with the current study, where relevant changes on eye and ear-base temperatures were observed the day after pigs were challenged, and they indicated illness signs several hours (4–6) before than clinical scores.

HR measurement has also been used as an indication of illness in animals [33,34]. The current study showed a relevant increase in the HR obtained remotely from “sick” animals during the day after being challenged with the infection, when compared to the changes in the HR of “healthy” pigs during the same period. Moreover, the significant difference of HR (*p* < 0.05) between “sick” and “healthy” animals started to be observed 4–6 h before the clinical observations indicated clear signs in “sick” animals. These results are supported by several studies, which have observed increased HR in animals presenting respiratory infections. For instance, Weingartl et al. [35] and Geisbert et al. [36] reported fever, tachycardia, and elevated RR as some of the first signs presented by horses with respiratory disease, after being inoculated with HeV. In addition, Reinhold et al. [37] showed that calves affected by *C. psittaci* infection increased their HR up to 160%, compared to the baseline.

Investigations related to respiratory diseases in pigs and other animals have also used RR as one of the relevant signs for the diagnosis of these diseases [3,38]. The preliminary results obtained in the present study showed that the average of RR remotely obtained from “sick” and “healthy” animals were similar during the first two observations performed the day after the challenge. During the next observations, the “sick” group showed higher average of RR than the “healthy” group (*p* > 0.05). Finally, “sick” animals showed an average of respiration rate 10 breath/min higher than “healthy” pigs in the last observation period (*p* < 0.05). These results could be related to the results reported by Kerr et al. [3], who did not observe correlation between RR and calcitonin receptor (CTR) when using CTR as a sign of APP infection. Van Reeth et al. [38] found increased RR in pigs affected by influenza, 24 h after being challenged with H1N2 virus. The fact that these preliminary results did not show an earlier significant difference of RR between groups could be related to the sensitivity of the method used or to slower changes in RR led by APP infection, compared to changes in HR and temperature.

The results observed in this pilot study could indicate a possible implementation of computer vision techniques to remotely monitor pigs, and to detect APP infections at an early stage. Pigs have previously been observed to start showing clear symptoms of APP after 11–23 h of being artificially infected, and the death of infected pigs has been observed from within a similar period (15–22 h after being infected) [3,24]. As severe cases and death appear to occur a short time after the clinical symptoms are identified, the detection of infected animals 4–6 h prior to the observation of clear symptoms, which was reported in the present study, could increase the success of the treatment, and decrease the rate of severe cases and death in pigs. This indicates that further research is needed to develop these techniques for on-farm use.

This pilot study had limitations due to the fact that cameras were held close to the animal for short periods to obtain the data; the fact that RR was analysed from non-radiometric infrared videos, which could have affected the sensitivity of the measurements due to the changes of light conditions; the fact that animal were identified manually; and the fact that a low number of the inoculated pigs were affected by the disease. In addition, pigs were inoculated with the disease, so the time between infection and clinical signs was predictable. The next step of this research will be focused on obtaining observations from cameras above the pen in a commercial piggery environment and analysing images of free-moving pigs that may become infected in a normal on-farm environment, incorporating TIR videos to measure respiration rate through temperature changes, and developing feature tracking algorithms to identify and track individual pigs for data analysis.

## 5. Conclusions and Further Research

Remotely sensed imagery and computer-based techniques showed great promise in early detection of *Actinobacillus pleuropneumoniae* infection in pigs. The changes in eye temperature, ear-base temperature and heart rate remotely obtained showed clear differences between sick and healthy pigs during the period evaluated. Where the group of sick animals exhibited a noticeable increase in these parameters, there were only slight increase in the healthy animals over the same period. Although respiration rate remotely obtained appeared to be significantly different between these two groups only in the last time point, further research is required to identify if the development of computer algorithms over thermal or RGB imagery could better assist with detection of changes in respiration rate related to illness in pigs.

During this pilot study, observations were taken while pigs were located within 1 m of the camera, rather than observing free-moving pigs. Further studies will obtain observations from cameras above the pen and analysing images of free-moving pigs. In addition, improvements in the described techniques and new techniques will be implemented in these studies to investigate the potential of several measures remotely obtained in the early detection of illness in pigs.

## Figures and Tables

**Table 1:** Clinical observations and scoring system used to identify animals with symptoms of *Actinobacillus pleuropneumoniae* (APP) infection.

Symptoms	Observations	Score Assigned
Nasal discharge	None	0
	Clear discharge	1
	Clear discharge for several observations	2
	Bloody frothy discharge from nose and mouth	3
Coughing	No coughing	0
	Coughs once	1
	Coughing episodes of 1–3 short coughs at a time	2
	Short coughs with signs of breathing difficulties	3
Laboured breathing	Normal (8–16 breath/minute)	0
	Breathing increased (>16 breaths/min)	1
	Abdominal breathing	2
	Laboured breathing, breathing through mouth, head extended	3
Reddened conjuctiva—Cyanosis	Normal	0
	Reddened conjunctiva, pale appearance	1
	Slightly red about the nose and lips	2
	Blueness about tongue, lips, mucous membrane of the eyes, ears, legs and reddened skin	3
Lethargy	Alert and active	0
	Depressed, lack of response to verbal stimuli, disinclination to move about, ears laid back	1
	Recumbent position, reluctance to get up	2
	Will not get up even if prompted, dullness of eyes	3
Anorexia	Eats	0
	Does not eat	1
	Does not eat and evident empty gastrointestinal tract (tucked in), not observed drinking	2
	Roughness in coat, tucked in and extremely dehydrated	3
Fever (rectal temperature)	No fever (39.3 °C)	0
	Temp rise (39.4–39.9 °C)	1
	Temperature increase at second observation	2
	Increase of temp 40–42 °C	3
